# 851 Rural Burn Center’s Initial Experience with a New Bovine Collagen Dermal Matrix Containing Added Elastin

**DOI:** 10.1093/jbcr/iraf019.382

**Published:** 2025-04-01

**Authors:** Tait Olaveson, Joshua Summers

**Affiliations:** Burn Center at Eastern Idaho Regional Medical Center; Burn Center at Eastern Idaho Regional Medical Center

## Abstract

**Introduction:**

A new bovine collagen-elastin dermal matrix (CEM) was recently made available in the United States after more than 17 years of use in more than 60 countries globally. This report is a review of our initial experience. We will discuss the perceived ease of use including both application and dressing protocol. Additionally, we will discuss the outcomes and follow-ups available from our US-based practice.

**Methods:**

A retrospective review was conducted of the patients on our service cared for with CEM from initial use in December 2021 through September 2024. Cases were stratified into type of injury, depth of injury and size of injury and each case was evaluated for length of time from initial application of CEM to definitive closure with split thickness skin graft.

**Results:**

There was a total of 117 CEM cases identified over this time period. Thirty five cases (30 percent of the total cases) were burn injuries (all full-thickness) with an average total body surface area (TBSA) of 15%. Of the 82 remaining cases, 43 were traumatic wounds and 39 were chronic wounds. The sizes ranged from 5.5cm2 to 5,384cm2. When classified into small (< 50cm2), medium (51 – 500cm2) and large (>500cm2), 23% were small, 54% were medium and 23% were large. Over the reviewed period, time to definitive closure with STSG decreased as experience and confidence increased. At the beginning of the process, average time to STSG placement from CEM application was 12 days and in the most current year the time has decreased to 7 days (decrease of 5 days or 41%). Additionally, 23% of the total cases were done as a one-stage procedure with CEM being placed and immediately covered with a STSG.

The dressing protocol was easy for our staff with no significant changes in practice. Our patients were either placed in a non-adherent dressing and a negative pressure dressing or a non-adherent dressing with a standard slow-release silver dressing and covered with standard conventional dressing. CEM is especially conformable and is not sided so is extremely easy to use. Finally, time to definitive closure is superior compared to other dermal matrices previously used in our institution.

**Conclusions:**

In our practice, all indications are that the newly available collagen-elastin dermal matrix decreases time to final closure even allowing, in some cases, for definitive closure with STSG at the time of dermal matrix application. We did not find any deviation from standard of care with the dressing protocol eliminating the variable of dressing change complications or the need for additional staff training.

**Applicability of Research to Practice:**

We routinely use dermal matrices in our practice. The newly available, easy-to-use collagen-elastin dermal matrix with improved conformability and decreased time to definitive closure increases our options when caring for patients with complex wounds and burn injuries.

**Funding for the Study:**

N/A

